# The effect of curcumin capsule on the severity and duration of primary dysmenorrhea among students: A triple-blind randomized controlled trial in the West of Iran

**DOI:** 10.1016/j.eurox.2025.100427

**Published:** 2025-09-08

**Authors:** Sara Abdoli, Salman Khazaei, Maryam Mehrpooya, Farideh kazemi, Ensiyeh jenabi, Reyhane Yazdaniroshan

**Affiliations:** a Student Research Committee, Hamadan University of Medical Sciences, Hamadan, Iran; b Malayer School of Medical Science, Hamadan University of Medical Sciences, Hamadan, Iran; c Department of Epidemiology, Research Center for Health Sciences, Hamadan University of Medical Sciences, Hamadan, Iran; d Department of Clinical Pharmacy, School of Pharmacy, Medicinal Plants and Natural Products Research Center, Hamadan University of Medical Sciences, Hamadan, Iran; e Mother and Child Care Research Center, Institute of Health Sciences and Technologies, Avicenna Health Research Institute, Hamadan University of Medical Sciences, Hamadan, Iran; f Mother and Child Care Research Center, Institute of Health Sciences and Technologies, Hamadan University of Medical Sciences, Hamadan, Iran

**Keywords:** Primary dysmenorrhea, Students, Curcumin, Severity, Duration

## Abstract

**Background:**

The present study was conducted to evaluate the efficacy of curcumin capsules on the severity and duration of primary dysmenorrhea.

**Methods:**

A randomized controlled trial conducted among female students at Hamadan University of Medical Sciences who met predetermined inclusion criteria in 2024 at 34 participants per group. In the intervention group, participants received curcumin capsules containing 500 mg of curcumin. These capsules were ingested once daily over the course of two menstrual cycles. The control group was administered placebo capsules, which contained 500 mg of starch, with the method of administration mirroring that of the intervention group. Data collection tools were including demographic characteristics questionnaire, visual pain ruler, pain duration and multidimensional verbal scale.

**Results:**

after the intervention, a significant difference emerged (P < 0.001). In the intervention group, dysmenorrhea severity decreased significantly from a mean of 6.5–4.53 in the first menstrual cycle and further reduced to 3.44 in the second cycle. In contrast, the control group showed no significant change. The intervention group's improvement was consistent across both cycles, highlighting the effectiveness of the intervention in reducing dysmenorrhea severity (P < 0.001).

**Conclusion:**

curcumin capsules can be used as an effective and low-risk complementary treatment for reducing the severity and duration of primary dysmenorrhea.

## 1 Introduction

Primary dysmenorrhea is one of the most common gynecological disorders among women of reproductive age, characterized by uterine pain and cramping, which can significantly impact quality of life, work and academic productivity, and even mental health [1]. This condition typically begins a few hours before or at the onset of menstruation and lasts for 48–72 h, resulting from excessive prostaglandin production in the endometrial lining [2]. Furthermore, elevated prostaglandin levels reduce uterine blood flow and increase neural sensitivity to pain, exacerbating the symptoms of primary dysmenorrhea [3].

Common treatments for primary dysmenorrhea include non-steroidal anti-inflammatory drugs (NSAIDs) such as ibuprofen and naproxen, as well as hormonal therapies like oral contraceptives [4]. These medications alleviate pain and reduce uterine contractions by inhibiting prostaglandin synthesis or restoring hormonal balance [5]. However, long-term use of these drugs may lead to adverse effects, including gastrointestinal complications, increased cardiovascular risks, and hormonal disruptions. Consequently, natural and complementary therapies have gained attention as lower-risk alternatives [6], [7].

Curcumin, the active compound in turmeric, is one such phytochemical that has attracted significant attention in recent studies due to its anti-inflammatory, antioxidant, and analgesic properties [8]. By inhibiting the cyclooxygenase-2 (COX-2) enzyme, which plays a key role in prostaglandin synthesis, curcumin can reduce inflammation and the severity of uterine contractions [9]. Additionally, studies have demonstrated that this compound can alleviate menstrual pain by modulating nitric oxide levels and improving uterine blood flow [10], [11].

Furthermore, curcumin exhibits phytoestrogen-like properties, enabling it to contribute to hormonal balance regulation and the reduction of dysmenorrhea symptoms [12]. Phytoestrogens are plant-derived compounds structurally similar to 17-beta estradiol, allowing them to bind to estrogen receptors and exert estrogenic effects [13]. These compounds are found in certain foods and medicinal plants, including soy, flaxseeds, red clover, and turmeric [14].

The phytoestrogens can modulate estrogen levels in the body, influencing hormonal function and reducing the severity of uterine contractions [15]. Many phytoestrogens possess potent antioxidant properties that may prevent cellular damage caused by oxidative stress. Additionally, certain phytoestrogens can decrease the production of inflammatory prostaglandins, thereby contributing to reduced menstrual pain [16].

Given the need for safer and lower-risk therapeutic approaches for managing primary dysmenorrhea, attention to herbal and complementary treatments, including curcumin, is warranted. Although numerous studies have investigated the anti-inflammatory and analgesic properties of curcumin, evidence regarding its specific effects on the intensity and duration of primary dysmenorrhea remains limited. Therefore, the present study was conducted to evaluate the efficacy of curcumin capsules on the severity and duration of primary dysmenorrhea. The findings of this research may contribute to a better understanding of curcumin's mechanisms of action in reducing menstrual pain and provide a natural, effective strategy for improving women's quality of life.

## 2 Materials and methods

### 2.1 Study type and participants

The present study was a triple-blind randomized controlled trial conducted among female students at Hamadan University of Medical Sciences who met predetermined inclusion criteria from 20 April 2024 until 16 December 2024.

The inclusion criteria included willingness to participate in the study, being single, having regular menstruation with intervals ranging from 21 to 35 days, absence of chronic diseases or genital tract disorders, no history of ovarian cysts, no known allergies to herbal medicine, absence of specific treatments aimed at alleviating dysmenorrhea symptoms in the preceding three months, being aged between 18 and 24 years, and exhibiting moderate to severe primary dysmenorrhea severity as assessed by a visual pain scale (score greater than 3). Additionally, participants were required not to engage in professional exercise and to have experienced stressors such as the loss of a parent within the past six months. The exclusion criteria included intolerance or sensitivity to the medication, and the use of any other herbal or pharmaceutical treatments during the intervention period.

### 2.2 Sample size

The sample size for this study was determined based on the pain intensity variable, utilizing the means comparison formula within Stata-14 software. Drawing from the findings of Tabari et al. [17] regarding the pain intensity variable in the intervention group, the pre-intervention mean (m) was 5.7 with a standard deviation (SD) of 1.52, while the post-intervention mean was 4.6 with an SD of 1.50. Adopting a two-sided alpha level of 0.05 and a power of 85 %, the calculated sample size was established at 34 participants per group. Moreover, accounting for a 10 % attrition rate, the final sample size was adjusted to 37 participants in each group.

### 2.3 Sampling

Upon introducing himself to the participants, the researcher elucidated the study's objectives and methodology, subsequently obtaining written informed consent from those willing to partake in the research. All participants were assured of the voluntary nature of their involvement and were informed of their right to withdraw from the study without any restrictions and at any stage of the intervention.

Initially, participants completed a questionnaire assessing their demographic and obstetric characteristics, as well as pain intensity, using the multidimensional verbal scale and the McGill pain ruler. This was employed to determine daily pain intensity, which facilitated the selection of individuals with moderate or severe dysmenorrhea. In the intervention group, the medication was administered from two days prior to the onset of menstruation through three days following the onset of bleeding, continuing this regimen for two menstrual cycles. Pain intensity was measured using a visual pain ruler immediately post-intervention and one month later. Additionally, participants completed the multidimensional verbal scale questionnaire at both of these time points.

In this study, participants were monitored over a three-day period (during admission, on the first day, and on the third day following medication administration). At each follow-up, assessments were made regarding drug tolerance, symptom alleviation, and any complications, including headaches and blurred vision. During the treatment phase, follow-up was conducted via telephone after medication administration in first menstrual cycle and again in second menstrual cycle to evaluate adherence to the treatment protocol, complete the questionnaire, and address any participant inquiries.

### 2.4 Randomization

In this study, 93 girls assessed for eligibility. Among them, 19 girls were excluded (6 due to chronic physical illness and 13 due to ovarian cyst). Participants were randomly assigned in a 1:1 ratio to two groups of 37 individuals, utilizing the Excel computer program with the command Choose randbetween (1;2) to allocate curcumin capsules and placebo capsules. The sequence of allocation was established prior to the commencement of the study by an individual not affiliated with the research team. To ensure allocation concealment, all drugs and placebos were placed in opaque, sealed envelopes and sequentially numbered. Consequently, participants received their respective envelopes according to their order of entry into the study.

### 2.5 Intervention

In the intervention group, participants received curcumin capsules (content of curcumin granules manufactured by Karen Pharmaceutical Company) containing 500 mg of curcumin, which were administered starting two days prior to the onset of menstrual bleeding and continued until three days after. These capsules were ingested once daily over the course of two menstrual cycles. Patients were recommended to consume the curcumin capsules with their meals. The control group was administered placebo capsules, which contained 500 mg of starch, with the method of administration mirroring that of the intervention group.

### 2.6 Data collection tools

• Demographic Characteristics Questionnaire: The demographic questionnaire encompasses various parameters, including age, parental occupation and education, and age of menarche, among others.
• Visual Pain Ruler: The McGill Pain Ruler is utilized to evaluate pain severity, categorized on a scale ranging from zero, indicating no pain, to ten, denoting severe pain. Han et al. [18]and Bekhradi et al. [19] assessed the validity and reliability of this instrument. The McGill Pain Ruler has been extensively employed in research, and according to Ngata et al., it is regarded as one of the most effective and reliable tools for measuring pain [20].
• Pain Duration: duration of dysmenorrhea (hour) in pre-intervention and post intervention in the first and second menstrual cycles was added to questionnaire, which participants complete through self-report.
• Multidimensional Verbal Scale: This scale comprises four levels. Level zero indicates the absence of painful menstruation, which does not interfere with daily activities. Level 1 denotes menstruation with mild pain that infrequently disrupts daily activities and requires minimal analgesics. Level 2 reflects moderate pain intensity that disrupts daily activities but does not necessitate missing school or work. Level 3 signifies severe pain that renders the individual unable to engage in daily activities, accompanied by significant systemic symptoms. Ozgoli et al. and Khodakarami et al. assessed the validity and reliability of this questionnaire[21], [22].

### 2.7 Ethical

Prior to participation in the study, all women provided written informed consent. The study was approved by the Ethics Committee of Hamadan University of Medical Sciences (Approval ID: 1402120810750.) and registered in the Iranian Clinical Trial Database (Registration ID: IRCT20180707040370N10).

### 2.8 Statistical analysis

The distribution of qualitative and quantitative variables is presented based on the study groups in terms of frequency (percentage) and mean (standard deviation), and they were tested using chi-square and independent t test as appropriate. Following data collection, the normality of the quantitative data was assessed separately for each group utilizing the Kolmogorov-Smirnov test. The independent *t*-test was used before the intervention and Analysis of covariance (ANCOVA) after the intervention in the first and second menstrual cycles by adjusting baseline values to compare the severity pain and duration, which had a normal distribution. For outcomes assessed at multiple time points following the intervention, repeated measures analysis of variance was utilized. A significance threshold of less than 0.05 was adopted. Statistical analyses were performed using Stata-14 software.

## 3 Results

**Fig. 1:**
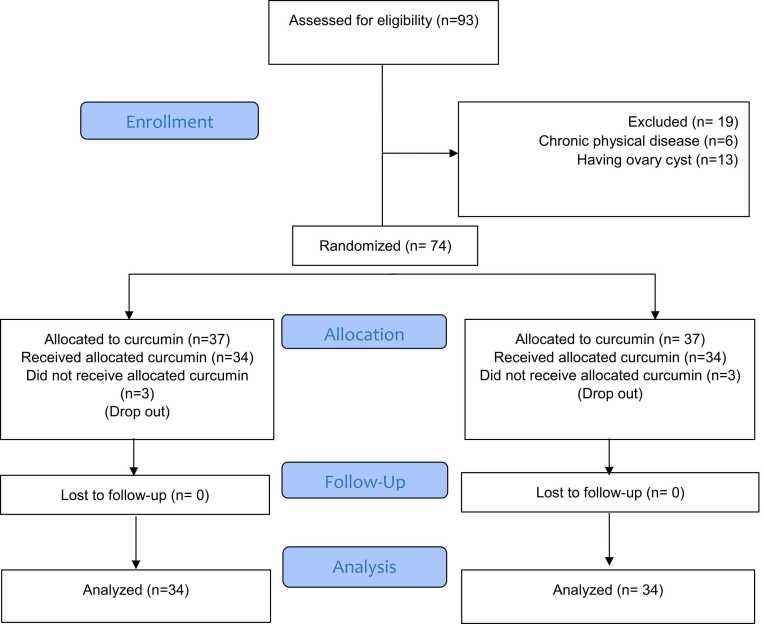
Flowchart of the present study design.

In total, 74 patients participated in this study including 34 patients in each of intervention (curcumin) and control (placebo) groups. At the beginning of the study, three participants in each group had not received the allocated treatment and were withdrawn from the study before the start of the follow-up phase. Fig. 1 presents the flowchart of the study process.

The demographic characteristics of participants in both the intervention and control groups were compared in Table 1. The results showed no significant differences between the two groups for age (P = 0.85), BMI (P = 0.72), age of onset of menstruation (P = 0.13), age of onset of dysmenorrhea (P = 0.72) and duration of bleeding (P = 0.71).

**Table 1:** Demographic characteristics of participants in two groups in baseline.

**Variable**	**Intervention group** **Mean±SD** **(n = 34)**	**Control group** **Mean±SD** **(n = 34)**	**P-Value**^a^
Age (year)	21.09 ± 1.77	21.18 ± 1.71	0.85
BMI (kg/m^2^)	22.33 ± 3.07	22.03 ± 3.90	0.72
Age of onset of menstruation (year)	12.91 ± 1.30	12.35 ± 1.59	0.13
Age of onset of dysmenorrhea (year)	14.03 ± 1.31	13.91 ± 1.36	0.72
Duration of bleeding	3.84 ± 0.85	3.76 ± 0.89	0.71

a Independent sample test.

The demographic characteristics and menstrual characteristics and lifestyle habits of participants' parents were compared between the intervention and control groups in Table 2. Significant differences were observed in mother's education (P = 0.003), father's education (P = 0.008), and father's occupation (P = 0.005). However, no significant difference was found in mother's occupation (P = 0.23). The menstrual characteristics and lifestyle habits of participants in both the intervention and control groups were compared. No significant differences were found between the groups for the volume of bleeding (P = 0.49), absence from class (P = 0.43), painful menstruation (P = 0.09) or physical activity (P = 0.08). However, participants in the intervention group more frequently experienced dysmenorrhea two days before menstruation compared to the control group (P = 0.13).

**Table 2:** Demographic characteristics of participants in two groups in baseline.

**Variable**		**Intervention group** **N (%)**	**Control group** **N (%)**	**P-Value**
**Demographic variables**				
Mother education	Primary school	0	8 (23.53)	**0.003**^a^
	High school	10 (29.41)	15 (44.12)	
	Diploma	13 (38.63)	6 (17.65)	
	Academic	11 (32.35)	4 (14.71)	
Mother occupation	House keeper	25 (73.53)	29 (85.29)	0.23^b^
	Employee	9 (26.47)	5 (14.71)	
Father education	Primary school	0	8 (23.53)	**0.008**^a^
	High school	9 (26.47)	12 (35.29)	
	Diploma	12 (35.29)	8 (23.53)	
	Academic	13 (38.24)	6 (17.65)	
Father occupation	Self-employee	27 (79.41)	21 (61.76)	**0.005**^a^
	Employee	7 (20.59)	4 (11.76)	
	Manual worker	0	9 (26.47)	
Menstrual characteristics and lifestyle habits				
The volume of bleeding	Mild	6 (17.65)	3 (8.82)	0.49^a^
	Medium	21 (61.76)	25 (73.53)	
	Sever	7 (20.59)	6 (17.65)	
Painful menstruation	Some times	16 (47.06)	8 (23.53)	0.09^a^
	Most of the time	11 (32.35)	19 (55.88)	
	Always	7 (20.59)	7 (20.59)	
Onset of dysmenorrhea	Two days before	24 (70.59)	18 (52.94)	0.13^b^
	The bleeding may start	10 (29.41)	16 (47.06)	
Absent of class	Yes	9 (26.47)	12 (35.29)	0.43^b^
	No	25 (73.53)	22 (64.71)	
Physical activity	Yes	8 (23.53)	15 (44.12)	0.08^a^
	No	17 (50)	16 (47.06)	
	Occasionally	9 (26.47)	3 (8.82)	

a Chi-square tests; ^b^ Fisher's exact test

Before the intervention, participants in both the intervention and control groups were compared for their symptoms. Notably, significant differences were observed for nausea and vomiting, with the intervention group experiencing more severe symptoms compared to the control group (P = 0.02). However, no statistically significant differences were found for pain, fatigue, and other symptoms between the two groups (P > 0.05). After the intervention, significant improvements were observed in the intervention group for several symptoms during the first menstrual cycle, including pain (P < 0.001), nausea and vomiting (P = 0.035), lethargy (P = 0.017), diarrhea (P = 0.032), changes in the nervous system (P = 0.038), fainting (P = 0.049), and headaches (P < 0.001). In the second menstrual cycle, the intervention group continued to show improvements, with significant differences noted for pain (P = 0.03), nausea and vomiting (P = 0.004), lethargy (P < 0.001), changes in the nervous system (P = 0.003), fainting (P = 0.001), and headaches (P = 0.003), as well as fatigue (P = 0.04) (Table 3).

**Table 3:** Comparison of pre-intervention, post intervention in the first and second menstrual cycle’s symptoms of participants in two groups.

**Variable**		**Pre-intervention**			**Post intervention in the first menstrual cycle’s**			**Post intervention in second menstrual cycle’s**		
		**Intervention group** **N (%)**	**Control group** **N (%)**	**P-Value**^a^	**Intervention group** **N (%)**	**Control group** **N (%)**	**P-Value**^a^	**Intervention group** **N (%)**	**Control group** **N (%)**	**P-Value**^a^
Pain	Mild	3 (8.82)	3 (8.82)	0.2	11 (32.35)	2 (5.88)	**0.001**	12 (35.29)	4 (11.76)	**0.03**
	Moderate	10 (29.41)	17 (50)		15 (44.12)	9 (26.47)		13 (38.24)	12 (35.29)	
	Severe	21 (61.76)	14 (41.18)		8 (23.53)	23 (67.65)		9 (26.47)	18 (52.94)	
Fatigue	Mild	5 (14.71)	2 (5.88)	0.46	9 (26.47)	4 (11.76)	**0.036**	17 [50]	9 (26.47)	**0.04**
	Moderate	14 (41.18)	17 (50)		17 (50.0)	12 (35.29)		13 (38.24)	13 (38.24)	
	Severe	15 (44.12)	15 (44.12)		8 (23.53)	18 (52.94)		4 (11.76)	12 (35.29)	
Nausea and vomiting	Mild	5 (14.71)	1 (2.94)	**0.02**	6 (17.65)	4 (11.76)	**0.035**	14 (41.18)	6 (17.65)	**0.004**
	Moderate	8 (23.53)	18 (52.94)		22 (64.71)	14 (41.18)		13(41.18)	9 (26.47)	
	Severe	21 (61.76)	15 (44.12)		6 (17.65)	16 (47.06)		6 (17.65)	19 (55.88)	
Lethargy	Mild	3 (8.82)	2 (5.88)	0.74	6 (17.65)	1 (2.94)	**0.017**	18 (52.94)	6 (17.65)	**< 0.001**
	Moderate	16 (47.06)	14 (41.18)		20 (58.82)	15 (44.12)		13 (38.24)	10 (29.41)	
	Severe	15 (44.12)	18 (52.94)		8 (23.53)	18 (52.94)		3 (8.82)	18 (52.94)	
Diarrhea	Mild	2 (5.88)	2 (5.88)	0.60	9 (26.47)	4 (11.76)	**0.032**	14 (41.18)	6 (17.65)	**0.001**
	Moderate	12 (35.29)	15 (47.06)		18 (52.94)	13 (38.24)		16 (47.06)	9 (26.47)	
	Severe	20 (58.82)	16 (47.06)		7 (20.59)	17 (50)		4 (11.76)	19 (55.88)	
Changing nervous system	Mild	3 (8.82)	1 (2.94)	0.45	6 (17.65)	3 (8.82)	**0.038**	16 (47.06)	6 (17.65)	**0.003**
	Moderate	10 (29.41)	14 (41.18)		21 (61.76)	14 (41.18)		14 (41.18)	12 (35.29)	
	Severe	21 (61.76)	19 (55.88)		7 (20.59)	17 [50]		4 (11.76)	16 (47.06)	
Faint	Mild	3 (8.82)	1 (2.94)	0.1	8 (23.53)	4 (11.76)	**0.049**	13 (38.24)	7 (20.59)	**0.001**
	Moderate	8 (23.53)	16 (47.06)		16 (47.06)	10 (29.41)		17 (50.0)	9 (26.47)	
	Severe	23 (67.65)	17 (50)		10 (29.41)	20 (58.82)		4 (11.76)	18 (52.94)	
Headache	Mild	6 (17.65)	1 (2.94)	0.08	13 (38.24)	3 (8.82)	**< 0.001**	15 (44.12)	5 (14.71)	**0.003**
	Moderate	9 (26.47)	15 (44.12)		19 (55.88)	13 (38.24)		14 (41.18)	12 (35.29)	
	Severe	19 (55.88)	18 (52.94)		2 (5.88)	18 (52.94)		5 (14.71)	17 (50.0)	

a Chi-square tests

**Fig. 2:**
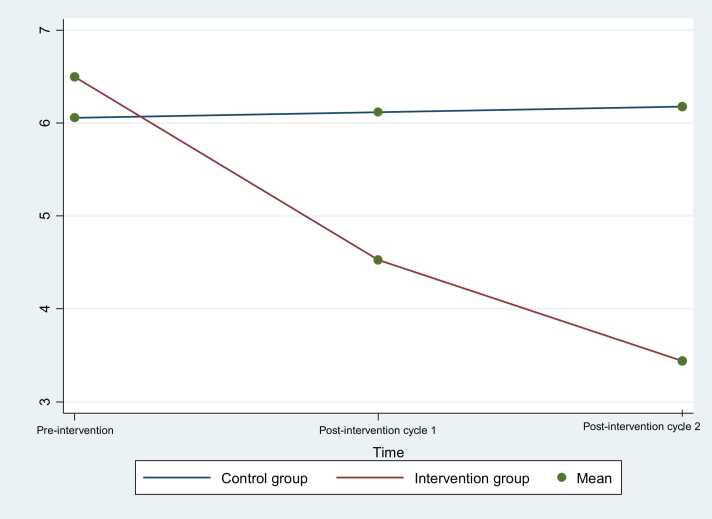
Severity of dysmenorrhea pre-intervention, post intervention in the first and second menstrual cycle’s symptoms.

**Fig. 3:**
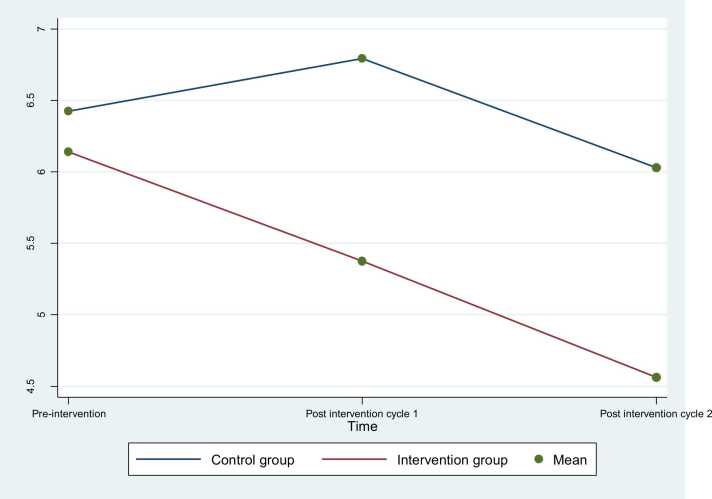
Duration of dysmenorrhea pre-intervention, post intervention in the first and second menstrual cycle’s symptoms.

The severity and duration of dysmenorrhea was compared before and after the intervention in both the intervention and control groups in Table 4**,** Fig. 2, Fig. 3. Initially, there was no significant difference in dysmenorrhea severity between the two groups (P = 0.21). However, after the intervention, a significant difference emerged (P < 0.001). In the intervention group, dysmenorrhea severity decreased significantly from a mean of 6.5–4.53 in the first menstrual cycle and further reduced to 3.44 in the second cycle. In contrast, the control group showed no significant change, with a slight increase from 6.06 to 6.11 in the first cycle and to 6.18 in the second cycle. The intervention group's improvement was consistent across both cycles, highlighting the effectiveness of the intervention in reducing dysmenorrhea severity (P < 0.001 for both treatment effect and time effect).

**Table 4:** Severity of painpre-intervention, post intervention in the first and second menstrual cycle’s symptoms.

**Variable**		**Intervention group** **(n = 32)**	**Control group** **(n = 34)**	**P-Value**
**Severity**	**Pre intervention**	6.5 ± 1.44	6.06 ± 1.34	0.21^a^
	**Post intervention in the first menstrual cycle’s**	4.53 ± 1.34	6.11 ± 1.3	**< 0.001**^b^
	**Post intervention in second menstrual cycle’s**	3.44 ± 0.88	6.18 ± 1.42	**< 0.001**^b^
	**P-Value**^C^	Treatment effect: P < 0.001, Time effect: P < 0.001		
**Duration (hour)**	**Pre intervention**	6.14 ± 0.98	6.24 ± 1.16	0.24^a^
	**Post intervention in the first menstrual cycle’s**	5.38 ± 0.91	6.79 ± 1.23	**< 0.001**^b^
	**Post intervention in second menstrual cycle’s**	4.56 ± 1.20	6.03 ± 1.03	**< 0.001**^b^
	**P-Value**^C^	Treatment effect: P < 0.001, Time effect: P < 0.001		

a Independent samples *t*-test

b ANCOVA with adjusting the baseline value.

c . Repeated measure ANOVA

The duration of dysmenorrhea in the intervention group showed significant reductions post-intervention. Initially, there was no significant difference between the intervention and control groups, with durations of 6.24 ± 1.16 and 6.14 ± 0.98 h, respectively (*p* = 0.24). However, after the intervention, the duration decreased to 5.38 ± 0.91 h in the first menstrual cycle and further reduced to 4.56 ± 1.20 h in the second cycle. In contrast, the control group experienced durations of 6.79 ± 1.23 and 6.03 ± 1.03 h in the first and second cycles, respectively. Both treatment and time effects were significant (*p* < 0.001) (Table 4).

## 4 Discussion

The results of the present study demonstrated that curcumin capsule supplementation significantly reduced both the severity and duration of primary dysmenorrhea.

These findings align with previous evidence regarding curcumin's anti-inflammatory, antispasmodic, and hormone-modulating properties. While conventional treatments such as NSAIDs and hormonal contraceptives remain effective, they are associated with adverse effects in some cases. In some cases, empiric gonadotropin-releasing hormone (GnRH) analog therapy may be offered. However, aside from potential side effects, their effectiveness in patients without endometriosis is not well understood [23]. Consequently, the search for natural and complementary therapies with comparable efficacy but fewer side effects has been a persistent focus of research.

A meta-analysis has demonstrated that curcumin supplementation can significantly reduce the severity of dysmenorrhea. During menstruation, prostaglandins, particularly PGF2α, are excessively secreted, leading to increased uterine contractions and reduced blood flow to uterine tissues. This condition can result in severe pain and tissue ischemia. Curcumin exerts its effects by inhibiting cyclooxygenase-2 (COX-2) enzyme activity, thereby decreasing prostaglandin production and subsequently reducing both uterine contractions and associated inflammation. This mechanism of action is similar to that of NSAIDs, but without inducing gastrointestinal adverse effects [24]. Given that elevated endometrial prostaglandin levels constitute the primary cause of intense uterine contractions in primary dysmenorrhea; this mechanism likely plays a crucial role in curcumin's therapeutic efficacy [25].In this context, Bahrami et al. (2022) conducted a clinical trial demonstrating that curcumin may positively influence serum IgE levels without significantly altering serum IL-10 and IL-12 concentrations in women with dysmenorrhea [26].Furthermore, a study investigating curcumin's effects on serum nitric oxide (NO) metabolites in young women with dysmenorrhea revealed that curcumin supplementation effectively regulated NO levels. Impaired blood flow to uterine muscles represents one of the key factors exacerbating dysmenorrhea [27]. Research indicates that curcumin may enhance NO production, promoting vasodilation and improving uterine blood perfusion. This effect could potentially alleviate ischemia and consequently reduce pain intensity [28].

Bahrami et al. (2021) reported a significant reduction in dysmenorrhea pain in both the curcumin and placebo groups, suggesting that curcumin exhibits effects comparable to placebo in alleviating premenstrual syndrome (PMS) and dysmenorrhea symptoms [29]. Pichardo et al. (2020), in a study evaluating curcumin’s efficacy for pain relief in primary dysmenorrhea, provided clinical evidence of its analgesic and anti-inflammatory properties [30].

A systematic review study aimed at investigating the effects and underlying mechanisms of turmeric and curcumin on PMS and dysmenorrhea reported that turmeric and curcumin influence PMS and dysmenorrhea through various mechanisms. They inhibit the production of prostaglandins, thereby reducing inflammation and pain. Furthermore, the antioxidant properties of curcumin protect against oxidative stress. Curcumin is a powerful antioxidant that can neutralize free radicals and reactive oxygen species, preventing cellular damage. Increased oxidative stress during menstruation can lead to neuro inflammation and enhanced pain receptor sensitivity. They also exhibit neurotransmitter-modulating properties, such as increasing serotonin and dopamine levels, which may contribute to their antidepressant and calming effects. Due to their anti-inflammatory and antioxidant properties, turmeric and curcumin have shown promising therapeutic effects in reducing PMS and dysmenorrhea symptoms [31].

The results of these studies collectively confirm that curcumin can be used as an effective and low-risk treatment for the management of primary dysmenorrhea. However, further studies are needed to conclusively confirm these findings.

### 4.1 Strengths and limitations

Adhering to all principles of clinical trials, including random allocation and allocation concealment, is one of the strengths of this study. The researcher, participant, and analyst were blinded to the allocation details. Standard questionnaires, all of which have undergone psychometric evaluation in Iran, were used in this research. Failure to control for other influencing factors such as lifestyle, diet, stress levels, and physical activity could impact the severity of dysmenorrhea, but these were not controlled for in this study.

## 5 Conclusion

Based on the findings of this study, curcumin capsules can be used as an effective and low-risk complementary treatment for reducing the severity and duration of primary dysmenorrhea in women. This effect is likely due to the inhibition of prostaglandin production, increased blood flow to the uterus, reduction of inflammation, and regulation of hormonal balance through the estrogen-like effects of curcumin. Given that conventional treatments such as NSAIDs and hormonal medications may be associated with various side effects, the use of herbal supplements like curcumin could be recommended as an alternative or adjunct approach in the management of menstrual pain.

## CRediT authorship contribution statement

**Abdoli sara:** Writing – review & editing, Writing – original draft, Visualization, Validation, Supervision, Project administration, Methodology, Investigation, Data curation. **Salman Khazaei:** Writing – review & editing, Writing – original draft, Validation, Software, Methodology, Formal analysis. **Maryam Mehrpooya:** Writing – review & editing, Writing – original draft, Methodology, Investigation. **Kazemi Farideh:** Writing – review & editing, Writing – original draft, Validation, Methodology, Investigation. **Jenabi Ensiyeh:** Writing – review & editing, Writing – original draft, Visualization, Validation, Supervision, Resources, Project administration, Methodology, Investigation, Funding acquisition. **Reyhane Yazdaniroshan:** Writing – review & editing, Investigation, Data curation.

## Funding

This study was funded by 10.13039/501100004697Hamadan University of Medical Sciences with Code: 1402120810750. The funder had no role in the design and conduct of the study.

## Declaration of Competing Interest

The authors declare no conflicts of interest.
